# Gene expression profiles of primary colorectal carcinomas, liver metastases, and carcinomatoses

**DOI:** 10.1186/1476-4598-6-2

**Published:** 2007-01-03

**Authors:** Kristine Kleivi, Guro E Lind, Chieu B Diep, Gunn I Meling, Lin T Brandal, Jahn M Nesland, Ola Myklebost, Torleiv O Rognum, Karl-Erik Giercksky, Rolf I Skotheim, Ragnhild A Lothe

**Affiliations:** 1 Department of Genetics, Institute for Cancer Research, Rikshospitalet-Radiumhospitalet Medical Center, Oslo, Norway; 2 Medical Biotechnology VTT, Turku, Finland; 3 Department of Cancer Prevention, Institute for Cancer Research, Rikshospitalet-Radiumhospitalet Medical Center, Oslo, Norway; 4 Surgical Department, Faculty Division Akershus University Hospital, Norway; 5 Division of Infectious Disease Control, Norwegian Institute of Public Health, Oslo, Norway; 6 Department of Pathology, Rikshospitalet-Radiumhospitalet Medical Center, Oslo, Norway; 7 Department of Tumor Biology, Institute for Cancer Research, Rikshospitalet-Radiumhospitalet Medical Center, Oslo, Norway; 8 Department of Molecular Biosciences, University of Oslo, Norway; 9 Institute of Forensic Medicine, Rikshospitalet-Radiumhospitalet Medical Center, Oslo, Norway; 10 Department of Surgical Oncology, Rikshospitalet-Radiumhospitalet Medical Center, Oslo, Norway

## Abstract

**Background:**

Despite the fact that metastases are the leading cause of colorectal cancer deaths, little is known about the underlying molecular changes in these advanced disease stages. Few have studied the overall gene expression levels in metastases from colorectal carcinomas, and so far, none has investigated the peritoneal carcinomatoses by use of DNA microarrays. Therefore, the aim of the present study is to investigate and compare the gene expression patterns of primary carcinomas (n = 18), liver metastases (n = 4), and carcinomatoses (n = 4), relative to normal samples from the large bowel.

**Results:**

Transcriptome profiles of colorectal cancer metastases independent of tumor site, as well as separate profiles associated with primary carcinomas, liver metastases, or peritoneal carcinomatoses, were assessed by use of Bayesian statistics. Gains of chromosome arm 5p are common in peritoneal carcinomatoses and several candidate genes (including *PTGER4*, *SKP2*, and *ZNF622*) mapping to this region were overexpressed in the tumors. Expression signatures stratified on *TP53 *mutation status were identified across all tumors regardless of stage. Furthermore, the gene expression levels for the *in vivo *tumors were compared with an *in vitro *model consisting of cell lines representing all three tumor stages established from one patient.

**Conclusion:**

By statistical analysis of gene expression data from primary colorectal carcinomas, liver metastases, and carcinomatoses, we are able to identify genetic patterns associated with the different stages of tumorigenesis.

## Background

Colorectal cancer (CRC) is the second most common cause of cancer related deaths in developed countries, including Norway [1,2]. Despite the fact that metastases are the leading cause of colorectal cancer deaths, the majority of genetic studies of colorectal carcinogenesis have focused on changes found in primary carcinomas, and the knowledge about the underlying molecular changes in more advanced disease stages remain limited. To obtain insights to this process, identification of molecular key events that distinguish primary from metastatic tumors is important. DNA microarray technology has become powerful for whole-genome investigations [3]. Recently, several reports have shown that results obtained by this technology can distinguish among subgroups of the same cancer tissue [4-7] as well as among different cancer types [8]. Additionally, genetic profiles have been identified that predict patients' clinical outcome in cancers of the breast, lung, central nervous system, digestive system, and prostate [9-15]. Several studies has investigated the expression profile of primary colorectal carcinomas [16]. However, only a few have investigated the gene profiles of lymph node and liver metastases derived from colorectal carcinomas [17-24], and so far none have studied metastasis to the peritoneal cavity by DNA microarrays. Whereas previous reports have focused only on the comparisons between normal mucosa and primary carcinomas, or primary carcinomas and metastases, we aimed to investigate the relationship between the primary carcinomas and metastases regardless of site, as well as the genetic patterns that might distinguish the different metastatic sites from each other. Therefore, we have analyzed the gene expression profiles of normal colon, primary carcinomas, liver metastases and peritoneal metastases, as well as an *in vitro *model of CRC progression by oligo microarrays, to compare the genetic patterns from the different stages of the colorectal tumorigenesis.

## Results

### Gene expression pattern in metastases versus those of primary tumors

**Figure 1:**
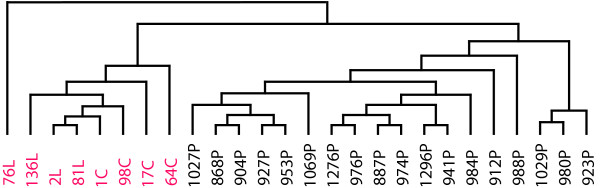
**Dendrogram from differentially expressed genes between metastases and primary tumors**. Dendrogram from hierarchical clustering of the 89 most statistical differentially expressed genes between metastases (n = 8; carcinomatoses and liver metastases together indicated in red) and primary carcinomas (n = 18 indicated in black), with a more than two-fold change derived from BAMarray.

**Figure 2:**
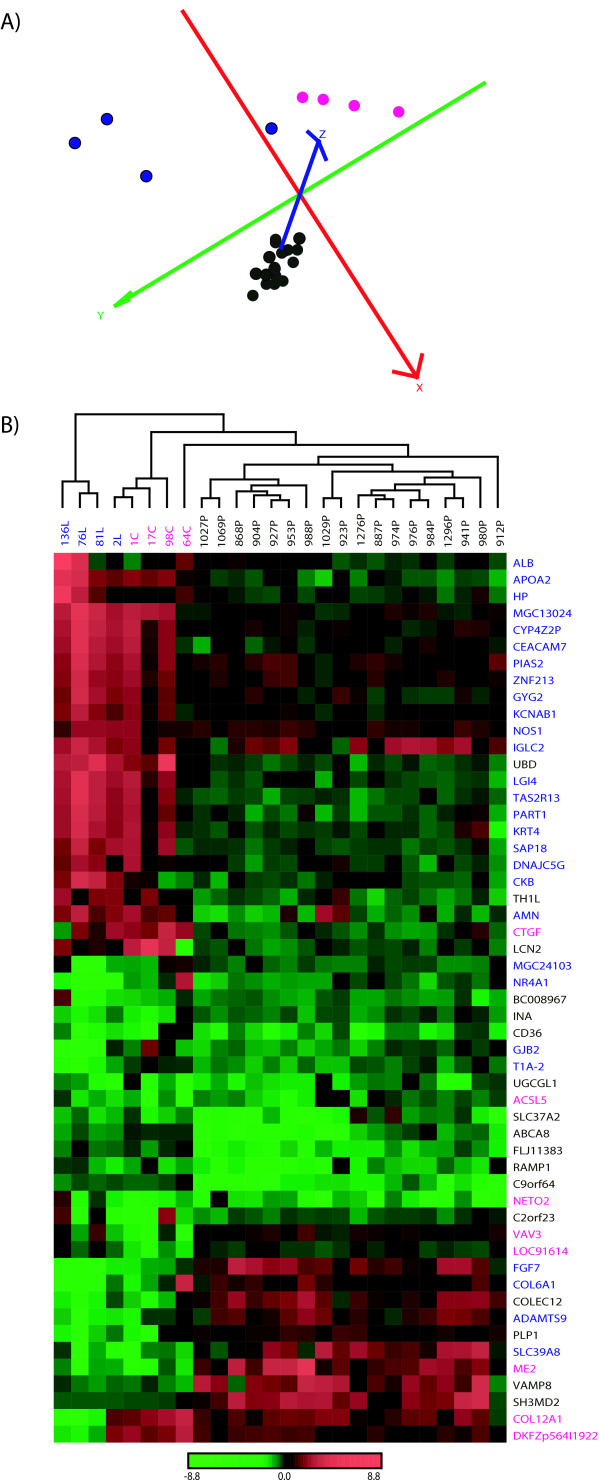
**Cluster analysis of differentially expressed genes between primary carcinomas, liver metastases and carcinomatoses**. A) PCA of the 53 most statistical differentially expressed genes between of primary carcinomas (n = 18, black), liver metastases (n = 4, blue), and carcinomatoses (n = 4, pink) expressed over three-fold derived from BAMarray. B) HCA of the same genes, with the same color coding. Genes are colored based on association to tumor site.

In order to find a gene expression pattern that distinguishes metastatic tumors from primary carcinomas, differentially expressed genes between metastases independent of site and primary carcinomas were identified. BAMarray [25] was used with a posterior variance between 0.92 and 1.06. The hundred most statistically significant genes associated with metastases (n = 8, liver metastases and carcinomatoses) and primary carcinomas (n = 18) were chosen, with a Z-cut absolute values ranged from 4.41 to 2.84 for metastases and 3.77 to 2.32 for primary carcinomas. Among these genes, 89 were expressed more than two-fold differently between the groups (twenty of these more than three-fold). Forty of the 89 genes were associated with the metastasis group, and thus, 49 with the primary group [see Additional file 1]. By using the 89 genes found from BAMarray, primary carcinomas and liver metastases were distinguished by hierarchical clustering (Figure 1). Liver metastases and carcinomatoses were intermingled, with the exception of one liver metastasis (76L) that is seen as an outlier compared to the rest of the metastases group. The gene expression profiles of three primary carcinomas (984P, 1029P, and 1296P) that later developed metastases did not show any similarity with each other or with the metastasis group when clustered on these selected genes. To find differentially expressed genes that distinguish the two metastatic sites from each other, as wells as from primary carcinomas, the dataset was grouped into primary carcinomas, liver metastases and carcinomatoses and further analyzed by BAMarray. A posterior variance between 0.93 and 1.19 were chosen, providing 51 genes associated with carcinomatoses, with absolute Z-cut from 3.59 to 2.30. Twenty-nine of these 51 genes were expressed more than two-fold compared to normal mucosa (Table 2). For primary carcinomas and liver metastases the hundred most statistically significant genes for each group derived from BAMarray were chosen, with absolute Z-cut at 4.15 to 2.95 for liver metastases, and 3.79 to 2.40 for primary carcinomas. Altogether, 251 differentially expressed genes from the three different tumor stages were chosen, and 53 of these genes revealed an expression level above three-fold in the median of the tumor stages (17 genes were associated with primary carcinomas, 28 with liver metastases, and eight with carcinomatoses), and among these, 23 genes were expressed above four-fold. To visualize the difference of the most statistically significant genes associated with each tumor site we performed PCA and HCA on the 53 genes derived from primary carcinomas, liver metastases, and carcinomatoses with expression above three-fold (Figure 2). The PCA plot distinguishes the three tumor stages from each other based on this gene list, except for one liver metastasis (2L) that shows a closer association to the carcinomatoses than to the other tumors (Figure 2A). These results were confirmed by HCA, where the dendrogram distinguishes seven out of the eight metastatic tumors from all of the primary carcinomas (Figure 2B). Three of four liver metastases clustered together, while 2L clustered in close association with the carcinomatoses as seen by PCA. One carcinomatosis (64C) appeared alone. We did not find a specific expression pattern of any of the genes in the selected gene list within the primary carcinoma group stratified by localization, Dukes' status, *TP53 *mutation status, or recurrence.

**Table 1:** Clinicopathological information.

***Tumor***	***Tumor ID***	***Dukes' stage*^*a*^**	***TP53 mutation status*^*b*^**	***Sex*^*c*^**	***Age*^*d*^**
primary carcinomas	923P	C	wildtype	M	85
	974P	B	ex8, c273, CGT→CAT, Arg→His	M	73
	980P	C	wildtype	F	75
	984P	C	wildtype	F	88
	988P	B	wildtype	F	66
	1029P	C	wildtype	M	83
	1069P	B	wildtype	M	74
	887P	B	wildtype	F	82
	927P	B	ex6, c190, CCT→CTT, Pro→Leu	F	73
	953P	B	ex6, 5 bp insertion; c216–217: GTG GTG to GTGgtggtGTG	M	68
	976P	B	wildtype	M	58
	1027P	B	ex7, c241–242, TCCTGC→TTCCGC, Ser-Cys→Phe-Arg	M	79
	868P	B	wildtype	M	64
	904P	B	ex8, c272, CTG→ATG, Val→Met	M	78
	912P	B	wildtype	F	66
	941P	B	ex8, c282, CGG→TGG, Arg→Trp	M	78
	1276P	B	wildtype	M	79
	1296P	B	ex7, c244, GGC→GTC, Gly→Val	M	76
liver metastases	136L	D	ex5, c132, AAG→AGG, Lys→Arg	M	68
	81L	D	wildtype	M	74
	2L	C	wildtype	M	75
	76L	D	ex7, c241, TCC→TC, 1 bp deletion	M	55
carcinomatoses	98C	D	wildtype	M	72
	1C	D	wildtype	F	62
	17C	C	ex5, c175, CGC→CAC, Arg→His	F	67
	64C	D	wildtype	M	40

^a^Dukes' stage of the primary tumors, and the primary tumor of liver metastases and carcinomatoses. ^b^ex, exon; c, codon; bp, base pair. ^c^M, male; F, female. ^d^Age at diagnosis.

**Table 2:** Genes (n = 29) associated with colorectal carcinomatoses as compared to primary tumors and liver metastases.

***Genebank Acc***.	***Gene Symbol***	***Gene Name***	***Z-cut***	***Fold change liver***	***Fold change carcinomatoses***	***Fold change primary***	***Relative difference, carcinomatosis vs. primary***
BC035498	*CCNE1*	cyclin E1	-3,59	-1.51	-2.15	1.05	2.24
AB011124	*ProSAPiP1*	ProSAPiP1 protein	3,24	1.37	2.26	1.28	1.77
NM_022772	*EPS8L2*	EPS8-like 2	-3,16	-1.64	-2.28	1.29	1.74
AK025824	*EPS8L2*	EPS8-like 2	-3,12	-1.63	-2.12	1.22	1.74
BC005245	*C1orf41*	chromosome 1 open reading frame 41	-3,07	-1.40	-2.63	-1.35	1.88
NM_017515	***SLC35F2***	solute carrier family 35, member F2	-2,89	-1.48	-2.75	-1.31	1.08
U73778	*COL12A1*	collagen, type XII, alpha 1	2,85	-1.72	2.34	1.15	1.77
BC004260	*CAPN10*	calpain 10	-2,85	4.54	-4.09	-2.34	2.03
NM_033018	*PCTK1*	PCTAIRE protein kinase 1	2,84	1.88	2.51	1.50	1.66
AK096896	***ASB12***	ankyrin repeat and SOCS box-containing 12	2,82	1.68	2.00	1.70	1.18
NM_033254	***BOC***	brother of CDO	2,81	1.26	2.09	1.30	1.61
NM_018043	*TMEM16A*	transmembrane protein 16A	2,78	-1.92	2.68	-1.84	5.08
BC012915	*MPRP-1*	metalloprotease related protein 1	-2,76	-1.70	-2.18	-1.57	1.39
BC002728	*THRA*	thyroid hormone receptor, alpha (erythroblastic leukemia viral (v-erb-a) oncogene homolog, avian)	-2,73	-1.41	-2.15	-1.23	1.73
X06482	***HBQ1***	hemoglobin, theta 1	2,71	1.69	2.61	1.28	2.09
X78947	*CTGF*	connective tissue growth factor	2,65	2.32	3.94	1.85	2.22
AF067817	*VAV3*	vav 3 oncogene	-2,63	-1.79	-2.50	-1.29	4.14
U86602	*EBNA1BP2*	EBNA1 binding protein 2	-2,63	-1.19	-4.81	-1.16	1.94
AL834404	*NETO2*	neuropilin (NRP) and tolloid (TLL)-like 2	-2,59	-1.96	-4.33	-1.47	2.93
M94065	***DHODH***	dihydroorotate dehydrogenase	-2,58	-1.63	-2.17	-1.04	2.08
NM_025109	*MYOHD1*	myosin head domain containing 1	-2,57	-1.68	-2.65	-1.03	2.55
NM_016234	*ACSL5*	acyl-CoA synthetase long-chain family member 5	-2,52	-2.52	-3.51	-1.52	2.07
NM_005132	***REC8L1***	REC8-like 1 (yeast)	-2,50	-1.41	-2.15	-1.11	1.19
NM_003412	*ZIC1*	Zic family member 1 (odd-paired homolog, Drosophila)	2,47	-1.90	2.53	-1.43	2.97
BC007300	*CHC1*	chromosome condensation 1	-2,47	-1.66	-2.78	-1.81	1.70
NM_139160	*DEPDC7*	DEP domain containing 7	-2,46	-1.07	-3.07	-1.15	2.66
NM_015419	*DKFZp564I1922*	adlican	2,45	-2.51	3.54	1.82	1.96
M55905	*ME2*	malic enzyme 2, NAD(+)-dependent, mitochondrial	-2,41	-2.10	-3.72	-1.53	2.20
NM_017744	*ST7L*	suppression of tumorigenicity 7 like	-2,33	-1.56	-2.11	-1.28	1.54

Z-cut is derived from BAMarray. Fold change; expression in fold change using medians of each group as compared to normal colonic tissue. Gene symbols in bold denote genes which are most dysregulated in the carcinomatosis cell line IS3, as compared to IS1 and IS2.

Genes located to chromosome arm 5p were of particular interest, as we have previously identified gain of 5p to be important for the CRCs' ability to metastasize to the peritoneal cavity [26]. Among the 115 genes at 5p in the dataset, 20 genes were more than two-fold higher expressed in carcinomatoses, as compared to liver metastases and primary carcinomas (Table 3).

**Table 3:** Genes (n = 20), located to chromosome arm 5p that are upregulated in carcinomatoses.

***Genebank Acc***	***Gene Symbol***	***Gene Name***	***Fold change carcinomatoses***	***Fold change liver***	***Folde change primary***	***Fold change carcinomatoses as compared to liver and primary***
L28175	*PTGER4*	Prostaglandin E receptor 4 (subtype EP4)	1.02	-4.41	-2.03	4.24
AK024116	*FLJ14054*	Hypothetical protein FLJ14054	1.20	-2.06	-3.46	3.96
AB061834	*RPL37*	Ribosomal protein L37	3.62	-1.02	1.04	3.61
BC000518	*BASP1*	Brain abundant, membrane attached signal protein 1	1.18	-1.96	-1.65	2.98
AF155135	*RAI14*	Retinoic acid induced 14	1.78	-1.35	-1.02	2.96
AF064876	*HCN1*	Hyperpolarization activated cyclic nucleotide-gated potassium channel 1	1.53	-1.31	-1.18	2.77
AK001989	***FLJ11127***	Hypothetical protein FLJ11127	1.25	-1.16	-1.52	2.58
BC008752	***ZNF622***	Zinc finger protein 622	1.29	-1.36	-1.05	2.49
AB020647	*FBXL7*	F-box and leucine-rich repeat protein 7	1.43	-1.03	-1.09	2.49
AK025310	*FLJ21657*	Hypothetical protein FLJ21657	1.07	-1.62	-1.15	2.45
U28043	***SLC9A3***	Solute carrier family 9 (sodium/hydrogen exchanger), isoform 3	1.01	-1.45	-1.39	2.43
BC001380	*SDHA*	Succinate dehydrogenase complex, subunit A, flavoprotein (Fp)	1.34	-1.04	-1.05	2.39
AF338650	*PDZK3*	PDZ domain containing 3	1.02	-1.68	-1.02	2.37
AB019494	*NIPBL*	Nipped-B homolog (Drosophila)	1.28	-1.13	-1.03	2.36
AF009301	*MARCH-VI*	Membrane-associated RING-CH protein VI	1.15	-1.32	-1.06	2.34
BC022339	*PC4*	Activated RNA polymerase II transcription cofactor 4	1.12	-1.28	-1.07	2.30
BC003353	*MGC5309*	Hypothetical protein MGC5309	1.15	-1.18	-1.08	2.27
AF189011	***RNASE3L***	Nuclear RNase III Drosha	1.04	-1.35	-1.07	2.26
BC017586	*MGC26610*	Hypothetical protein MGC26610	1.17	-1.08	-1.06	2.24
AY029177	*SKP2*	S-phase kinase-associated protein 2 (p45)	1.04	-1.00	-1.07	2.08

Ratios; expression in fold change using medians of each group as compared to normal colonic tissue. Fold change carcinomatoses; expression fold in carcinomatoses – (fold in liver metastases + primaries)/2.

Genes in bold are upregulated in the carcinomatoses cell line IS3.

**Figure 3:**
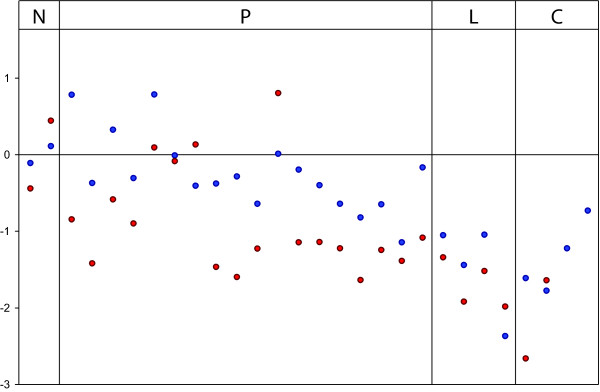
***ELAC1 *downregulation in metastases**. We used real-time RT-PCR to validate the expression of five genes with altered expression in metastases. *ELAC1 *was validated as a downregulated gene in colorectal cancer, with a particular downregulation in the liver metastases and carcinomatoses. Values are here normalized according to values from normal colon mucosa before log2-transformation. Red and blue colored circles denote results from individual samples using real time RT-PCR and microarray experiments, respectively. N, normal colon mucosa; P, primary carcinoma; L, liver metastasis; C, carcinomatosis.

We selected five of the genes with different expression levels between metastases and primary carcinomas for experimental validation by real-time RT-PCR. Out of these, three genes were validated as differentially expressed between the groups. These were upregulation of *TM4SF1 *and downregulation of *ELAC1 *(Figure 3) and *CCNE1 *in metastases. *CCNE1 *had particularly low expression in the carcinomatosis group. RT-PCR data of *INCENP *was only weakly following the same trend as the microarray data, whereas validation failed for *PIAS2*.

### Expression profile stratified by *TP53 *mutation status

Altogether, ten of 26 tumors harbor *TP53 *mutation in exons 5–8 (seven of 18 primary carcinomas, two of four liver metastases, and one of four carcinomatoses; Table 1). In order to investigate the influence of the *TP53 *mutation status on the gene expression signatures, BAMarray analysis was performed on all tumors dependent on *TP53 *mutation status. A posterior variance between 0.90 and 1.13 were used, and the hundred most differentially expressed genes (with statistical significance) both in the tumors with *TP53 *mutation (absolute Z-cut ranging from 3.49 to 2.41) and from those with wild type *TP53 *were chosen (absolute Z-cut 3.64 to 2.24). Among these two hundred genes, 75 were expressed more than two-fold differently between the groups (27 genes with expression level above 3.0). Of these 33 genes were associated with tumors harboring *TP53 *mutation, and 42 genes with those without [see Additional file 2]. PCA and HCA were performed on the 75 genes chosen from BAM analysis, and both analyses show a clear tendency to discriminate the tumors with *TP53 *mutation from those without, independently of stage [see Additional file 3]. In the same manner, the mutant *TP53 *primary tumors (n = 7) have been analyzed versus the wild type *TP53 *primary tumors (n = 11), and the gene lists associated with either group is overlapping with the ones found for all tumors stratified by *TP53 *mutation status.

### Cell line model

**Figure 4:**
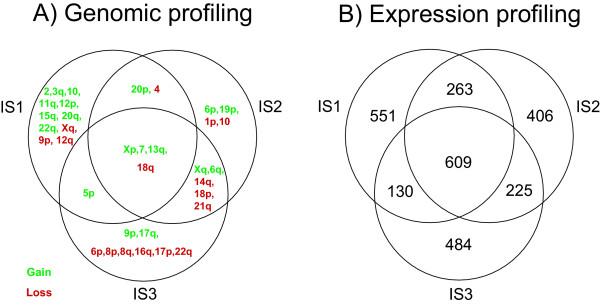
**Genome and transcriptome profiles of cell line model**. A) Genomic changes in three cell lines IS1, IS2, and IS3 from a primary carcinoma, its corresponding liver- and peritoneal metastases derived from the same patient. B) Genes expressed in fold change above 2.0 in the same cell lines. 609 genes are found in common between the three cell lines, whereas 263 genes are shared between IS1 and IS2, 130 genes in common between IS1 and IS3, and 225 genes are shared between the metastases cell lines, IS2 and IS3. 551- (IS1), 406- (IS2), and 484 genes (IS3) are only seen in one cell line.

The three cell lines IS1, IS2, and IS3 are derived from a primary carcinoma, liver metastasis, and carcinomatosis from the same patient. We have previously shown common and specific chromosomal changes for each of the cell lines [27] (Figure 4A). Here, we analyzed the gene expression profiles for the same cell lines. IS1 had 1553 genes, IS2 had 1503 genes, whereas IS3 had 1448 genes with an expression level above two-fold as compared to normal colonic mucosa. Among these genes, 609 genes were common in all the three cell lines, whereas IS1 and IS2 share 263 genes, and IS1 and IS3 share 130 genes. IS2 and IS3 share 225 genes with an expression above two-fold, which might be considered general metastasis genes independent of site (Figure 4B). Among the genes dysregulated more than two-fold in the three cell lines, we chose the 200 most dysregulated genes solely for each cell line. This resulted in a list of 600 genes associated with the different tumor stages (data not shown).

### Comparisons of *in vivo *tumors with *in vitro *model

To address whether the cell lines derived from the different stages are representative models of *in vivo *tumors, we performed hierarchical cluster analysis on the primary carcinomas (n = 18), liver metastases (n = 4), and carcinomatoses (n = 4), based on the most dysregulated genes found associated with each cell line [see Additional file 4]. Three of the four liver metastases cluster close to each other, whereas the carcinomatoses are spread among the primary tumors.

When comparing the most differentially expressed genes specific for *in vivo *tumors (primary carcinomas, liver metastases, and carcinomatoses; Figure 2) with the *in vitro *model, we found that 40 of 59 *in vivo *specific genes were regulated in the same direction in both cell lines and solid tumors. For the genes associated with liver metastasis, 19 of 28 genes were regulated in the same way in IS2. Five of the 28 genes were as well most dysregulated in IS2 as compared to IS1 and IS3. For the genes associated with carcinomatosis, 6 of 8 genes were confirmed in IS3 (2 of 8 genes are most dysregulated in IS3 compared to IS1 and IS2), and for the genes specific for primary carcinomas, 15 of 17 genes were confirmed in IS1 (4 of 17 genes are most dysregulated in IS1 compared to IS2 and IS3) (Table 4).

**Table 4:** Genes in common among *in vivo *tumors and *in vitro *cell lines.

***Genebank Acc***.	***Gene Symbol***	***Gene Name***	***Z-cut***	***Stage***	***Fold change IS1***	***Fold change IS2***	***Fold change IS3***
X78947	*CTGF*	connective tissue growth factor	2,65	C		1.11	1.59
AF067817	*VAV3*	vav 3 oncogene	-2,63	C	-24.77	-3.89	-1.23
AL834404	***NETO2***	neuropilin (NRP) and tolloid (TLL)-like 2	-2,59	C	1.82	1.21	-12.01
NM_016234	*ACSL5*	acyl-CoA synthetase long-chain family member 5	-2,52	C	-4.64	-1.75	-3.09
NM_139160	*LOC91614*	novel 58.3 KDA protein	-2,46	C	-2.23	-1.59	2.58
M55905	***ME2***	malic enzyme 2, NAD(+)-dependent, mitochondrial	-2,41	C	1.21	-1.80	-1.66
NM_000620	*NOS1*	nitric oxide synthase 1 (neuronal)	4,15	L	2.06	2.51	-1.69
NM_013317	***T1A-2***	lung type-I cell membrane-associated glycoprotein	-3,95	L	-9.86	-12.64	-2.37
AK097373	*CYP4Z2P*	cytochrome P450 4Z2 pseudogene	3,92	L	1.78	1.19	-15.97
X98311	*CEACAM7*	carcinoembryonic antigen-related cell adhesion molecule 7	3,92	L	1.59	1.41	-7.00
NM_139284	*LGI4*	leucine-rich repeat LGI family, member 4	3,86	L	2.12	1.55	-3.53
AF227137	*TAS2R13*	taste receptor, type 2, member 13	3,81	L	1.35	1.20	5.73
K00422	***HP***	haptoglobin	3,70	L	1.32	1.33	1.64
NM_001848	*COL6A1*	collagen, type VI, alpha 1	-3,62	L	-5.51	-39.77	-1.60
X04898	*APOA2*	apolipoprotein A-II	3,57	L	7.16	5.61	-1.18
BC016147	*NR4A1*	nuclear receptor subfamily 4, group A, member 1	-3,41	L	-6.69	-3.26	-6.91
NM_173650	***DNAJC5G***	DnaJ (Hsp40) homolog, subfamily C, member 5 gamma	3,37	L	2.20	2.70	-3.42
NM_152576	***MGC24103***	hypothetical protein MGC24103	-3,36	L	-9.78	-14.62	-1.10
AK056254	*KRT4*	keratin 4	3,36	L	2.17	1.34	-8.59
NM_004671	*PIAS2*	protein inhibitor of activated STAT, 2	3,29	L	1.96	1.19	2.16
AF328788	***AMN***	amnionless homolog (mouse)	3,12	L	2.44	3.06	-6.08
BC007287	*ZNF213*	zinc finger protein 213	3,07	L	3.11	1.81	-2.05
BC012125	*SLC39A8*	solute carrier family 39 (zinc transporter), member 8	-3,04	L	-4.10	-2.34	2.97
NM_020249	*ADAMTS9*	a disintegrin-like and metalloprotease (reprolysin type) with thrombospondin type 1 motif, 9	-2,98	L	1.03	-1.45	-2.91
M60828	*FGF7*	fibroblast growth factor 7 (keratinocyte growth factor)	-2,95	L	-7.06	-8.61	-15.00
M98398	***CD36***	CD36 antigen (collagen type I receptor, thrombospondin receptor)	-3,17	P	-23.00	-21.88	1.11
NM_033201	***BC008967***	hypothetical gene BC008967	-2,95	P	-7.38	-4.64	-2.15
BC001634	*VAMP8*	vesicle-associated membrane protein 8 (endobrevin)	-2,78	P	-1.78	-2.89	2.65
NM_022912	*C2orf23*	chromosome 2 open reading frame 23	-2,72	P	-4.00	-7.64	1.80
M27110	*PLP1*	proteolipid protein 1 (Pelizaeus-Merzbacher disease, spastic paraplegia 2, uncomplicated)	-2,67	P	-3.05	-4.03	1.94
AB038518	*COLEC12*	collectin sub-family member 12	-2,62	P	-9.65	-9.96	-10.22
AB020629	*ABCA8*	ATP-binding cassette, sub-family A (ABC1), member 8	-2,60	P	-1.85	-2.07	-6.80
Y12653	*UBD*	ubiquitin D	2,60	P	2.07	1.72	1.35
AK025416	***UGCGL1***	UDP-glucose ceramide glucosyltransferase-like 1	-2,60	P	-5.63	-3.92	1.81
AK021429	*SH3MD2*	SH3 multiple domains 2	-2,59	P	-1.07	-1.26	2.22
NM_032727	*INA*	internexin neuronal intermediate filament protein, alpha	-2,54	P	-2.19	-2.48	-1.06
AK074207	*SLC37A2*	solute carrier family 37 (glycerol-3-phosphate transporter), member 2	-2,50	P	-2.68	-2.25	1.38
AJ001014	*RAMP1*	receptor (calcitonin) activity modifying protein 1	-2,46	P	-6.00	-4.48	-44.44
AB007895	*FLJ11383*	hypothetical protein FLJ11383	-2,41	P	-1.11	1.09	2.92
NM_016397	***TH1L***	TH1-like (Drosophila)	2,40	P	2.39	2.35	-1.27

Z-cut is derived from BAMarray., L; liver metastases, C; carcinomatoses, P; primary carcinomas Fold change; expression in fold change as compared to normal colonic tissue. Genes shown in bold are most dysregulated in the corresponding cell line when compared to solid tumors.

When evaluating the genes associated with carcinomatosis from *in vivo *and *in vitro *(IS3) models, we found that 20 of the 29 genes defined from the *in vivo *data had the same type of alteration also in the cell line model (six of 29 genes were most dysregulated in IS3 compared to IS1 and IS2; Table 2). Among the upregulated genes on 5p in carcinomatoses (*in vivo *model), four genes showed the same type of alteration in the carcinomatosis cell line IS3 as compared to IS1 and IS2 (Table 3).

## Discussion

Several studies have investigated the expression profiles of human tumors taking advantage of the microarray technology, including some studies of primary colorectal carcinomas [16]. Despite the fact that metastases are the leading cause of CRC deaths, few have investigated the expression profiles of metastases, and the reports published have focused on lymph nodes and liver metastases from CRC [19-24,28,29]. Using 22k oligo microarrays we have nearly doubled the number of DNA sequences studied compared to most previous publications investigating gene expression levels of CRC metastases [18-21,24]. By comparing the genetic profile from different tumor stages of CRC, including primary tumors and two metastatic sites, liver and peritoneum, we were able to find potential genes associated with metastasis, which might play an important role in the metastatic process. By using Bayesian ANOVA for microarray [25], we were able to identify differentially expressed genes associated with the groups included. This method has its strengths when comparing more than two groups. Further statistical tools, such as HCA and PCA, visualize the differences in the gene expression between the different stages of CRC, as well as between the two metastatic sites, liver and the peritoneum (Figures 1 and 2). Tumors from the two metastatic sites reveal gene expression profiles more closely related to each other than to the primary carcinomas. We selected the primary samples in order to obtain a similar representation from the different topographical sites in colon and rectum, from patients from the intermediate clinical groups (Dukes' B and C). Thus, it seems reasonable to expect that the expression profiles of these are representative, supporting the findings of distinct profiles of the metastases.

### A general gene expression pattern for metastases

HCA and PCA were used to visualize the different transcript levels of 89 genes in primary tumors and metastases. Forty genes in this expression profile were specific for the metastasis group [see Additional file 1], including several genes previously reported in relation to cancer metastasis. Interestingly, most of the genes have not previously been described in colorectal metastases, and the genes of particular interest are involved in processes like apoptosis and cell growth. Among the downregulated genes are *CASP1, ELAC1*, *INCENP*, *ME2*, and *PLA2G2A*. CASP1 has been shown to induce apoptosis, and disruption of apoptotic pathways is in general an important factor in tumor development, and downregulation of this gene has also previously been reported in primary CRCs [30]. *ELAC1*, encoding an RNA processing enzyme, is located on the chromosome band 18q21, which chromosomal loss has previously been linked to poor prognosis in colorectal cancer [31]. The *ELAC1 *locus was targeted in a 300 kb homozygous deletion in lung cancer, which also involved the *ME2 *gene [32]. INCENP is required for correct chromosome segregation and cytokinesis during mitosis and complexes with Aurora B kinases [33]. Inhibition of *INCENP *is associated with chromosome aneuploidy, and downregulation of this gene might be important in metastases. Mice lacking expression of *PLA2G2A *have revealed increased colonic polyposis, and although gene mutations is not reported, lack of expression and sequence losses from this locus (chromosome band 1p36) are found in human colorectal carcinomas [34]. Interestingly, *TM4SF1*, a member of the transmembrane 4 superfamily, was upregulated in the metastases group. This antigen is known to be highly expressed in several cancer types, including CRC [22,35], and increased level of *TM4SF1 *has been associated with development of metastases and poor clinical outcome in patients with lung cancer [36].

Genes differentially expressed between primary CRCs and normal tissue have been reported by several studies [16], but only few have shown the differences in expression profiles between primary tumor and lymph node- and liver metastases. By statistical analyses we found 49 genes associated with primary carcinomas as compared with both liver metastases and carcinomatoses [see Additional file 1]. Among the genes with increased expression were *CDCA7, CXCL1, CXLC2, CXCL3*, and *LCN2*. Cell division cycle associated 7, *CDCA7*, upregulated among the primary carcinomas, is suggested to be involved in neoplastic transformation as it acts as a direct Myc target gene [37]. The chemokines *CXCL1, CXCL2*, and *CXCL3 *also called *GRO *oncogenes, are involved in angiogenesis, development, and homeostasis. Upregulation of *CXCL1 *[16,21,38-41] and *CXCL3 *[42] has previously been observed in CRCs and other cancer types [43]. LCN2 binds and transports small lipophilic molecules, and is involved in cell regulation [44]. Additionally, LCN2 acts as a subunit of the MMP-9 that has been observed in increased levels in tumor cells in the transition from colonic adenomas to carcinomas [45]. Among the downregulated genes in primary carcinomas were *AKR1B10*, *CD36*, and *LMNB1*. The expression of aldo-keto reductase (*AKR1B10*) and collagen receptor *CD36 *is highly reduced in the primary group, and is previously reported downregulated in CRCs [46]. *LMNB1 *belongs to the lamin family, where the proteins are involved in nuclear stability, chromatin structure and gene expression. Reduced expression have been seen in several cancer types, including CRC [47].

### Genes associated with liver metastases

By using BAMarray on expression profiles of liver metastases, in comparison with primary carcinomas and carcinomatoses, we identified the most statistically significant genes associated with liver metastases (Figure 2B). These genes might play a significant role in the metastasis to the liver. Several interesting genes were found downregulated, such as *ADAMTS9 *and *COL6A1 *in the liver metastasis group. *ADAMTS9*, a thrombospondin metalloproteinase, is a member of the ADAM-TS family, which controls organ shape during development, inhibit angiogenesis, and are implicated in cancer [48,49]. Recently, we have found another gene in the same family, *ADAMTS1*, to be a novel candidate for epigenetic inactivation by promoter hypermethylation in colorectal carcinomas [50]. *COL6A1 *belongs to a collagen family, and are previously reported upregulated in metastases from medulloblastoma and cancers of the breast and prostate [11]. Carcinoembryonic antigen-related cell adhesion molecule 7 (*CEACAM7*) is expressed in normal colon, but reported downregulated in adenomas and colorectal carcinomas [42,51]. Controversially, we found *CEACAM7 *upregulated in the liver metastases, suggesting another function in the metastatic tumors. Another gene with increased expression in liver metastases of particular interest was *PIAS2*. Protein inhibitor of activated STAT2 (*PIAS2*) is a transcription factor controlling cell cycle arrest after DNA damage through various cellular pathways [52], such as STAT-, MYC- and TP53 pathways, as transcriptional coregulators [53,54]. The conflicting RT-PCR and microarray data for *PIAS2 *may be due to their targeting of different mRNA splice variants. The *PIAS2 *microarray probe targets the exon-exon junction 12–13, whereas the RT-PCR primers target the exon-exon junction 5–6 of the transcript.

### Genes associated with peritoneal carcinomatoses

To our knowledge, only one molecular genetic study has previously been performed on carcinomatoses from colorectal cancer [26], and for the first time, carcinomatoses are investigated at the gene expression level. By using Bayesian ANOVA statistics we identified a gene pattern associated with carcinomatoses (Table 2, Figure 2). Of the 29 genes expressed above two-fold in the carcinomatosis group compared to primary carcinomas and liver metastases, several of the genes found were of interest in relation to cancer biology, such as the upregulation of *DKFZp564I1922 *(alias adlican), and *CTGF*, and the reduced expression of *CCNE1, CHC1*, and *MYOHD1*. The gene encoding the hypothetical protein adlican is previously seen highly expressed in colorectal cancer compared to normal tissue [39]. Expression studies of primary CRCs have observed dysregulation of several collagens [16,40,55-57]. *CTGF *is a connective tissue growth factor that promotes proliferation, and seems to play an important role in the metastatic process, as this gene has been associated with tumor progression in several types of cancer [58-61]. However, the expression of *CTGF *seems to play a varying role in several cancer metastases, as expression of this gene is also reported as a factor for better prognosis by suppression of tumor growth [62]. CCNE1 is an important component in the cell cycle regulation, and as a target in the carcinogenesis, overexpression over cyclin E has been observed in several tumor types [63-65]. However, decrease of *CCNE1 *from primary colorectal carcinomas to liver metastases is seen, and reduction of cyclin E in primary carcinomas is associated with poor prognosis and metastasis to the peritoneum [66]. This is in line with our observation, as CCNE1 showed a reduced expression level in peritoneal carcinomatoses compared to primary tumors. *CHC1 *is located at chromosome band 1p36 that is commonly deleted in CRC [67]. It binds to chromatin and is involved in the regulation of onset of chromosome condensation [68], thus reduced expression of this gene might lead to failure in the chromosome segregation. Several myosin genes are previously associated with metastasis [11], and interestingly, myosin head domain (*MYOHD1*) is found dysregulated in carcinomatoses and liver metastases in the present dataset.

By using genomic profiling techniques on different stages of the CRC progression, we have previously identified gain of 5p by DNA copy number alterations to be specific for the metastatic process to peritoneal cavity [26,27]. In this chromosomal region we found 20 genes upregulated in carcinomatoses as compared to the other stages (more than two-fold; Table 3), including *FBXL7, PTGER4, SKP2*, and *ZNF622*.

### *TP53 *gene profile

By using BAMarray, we distinguished the expression pattern of the tumors according to their *TP53 *mutation status. Mutations in *TP53 *are one of the most frequently encountered genetic alterations in human solid tumors. More than half of all primary CRCs carry a mutation within this gene, and inactivation of *TP53 *is believed to play a central role in the genetic tumor progression model [69]. Interestingly, there seem to be differences in the genetic pattern in tumors revealing mutation from those with wild type *TP53 *across the tumor stages [see Additional files 2 and 3], supporting the importance of *TP53 *mutation independent of CRC stage. Additionally, the same pattern is observed in the primary colorectal carcinomas. A similar pattern has been observed in breast carcinomas as tumors with *TP53 *mutation show a different gene expression profile than those without [70]. Taken together, these observations suggested that inactivation of *TP53*, indirectly or directly, leads to altered expression of the downstream genes.

### Comparison of *in vitro *models with *in vivo *tumors

The gene expression variations in the cell line model representing three different tumor stages: primary carcinomas, liver metastasis, and peritoneal metastasis from the same patient, provide clues to the understanding of the cancer progression process (Figure 4) [27]. We arranged the solid tumors by hierarchical clustering based on genes derived from the cell line model [see Additional file 4]. The *in vivo *tumors are on the dendrogram partly positioned into correct stages, but not as successfully as by using the genes derived from the *in vi*vo tumors themselves (Figure 2). Comparisons of the genetic patterns derived from analyses of the *in vivo *tumors with corresponding expression patterns from the cell line model reveal analogous expression changes of many genes, and thus strengthen our findings in the solid tumors (Tables 2, 3, and 4). However, the relationship between cell lines and *in vivo *tumors based on gene expression should be handled with caution. Comparisons of gene expression patterns in cell lines compared to their corresponding tumor tissue reveal similarities, and cell lines are thought to reflect the molecular signatures of the tissue from which the cell lines originated. Nevertheless, it has been shown that clustering algorithms separate cell lines from the *in vivo *tumors of the same cancer disease [71,72].

## Conclusion

By studying the gene expression of primary colorectal carcinomas, liver metastases and carcinomatoses, we were able to identify genetic patterns associated with each of the different stages. We emphasize the importance of the genetic profiles, where the combination of several genes is the key feature that is associated with the different stages of CRC. Several interesting candidate genes representing potentially therapeutic targets are found in the present data set. Validation of gene expression signatures in larger series needs to be performed to improve the understanding of the metastatic process of CRC further.

## Materials and methods

### Material

Altogether, 29 tissue samples were included in this study; three of these were from normal colon, eighteen primary colorectal carcinomas (14 Dukes' B and four Dukes' C; 8 from the right side of colon, 5 from the left side, and 5 from rectum), four liver metastases, and four peritoneal metastases (carcinomatoses). In addition, as an *in vitro *model for cancer progression, three cell lines derived from tumor samples of the same patient were included (Table 1). These were Isreco1 (IS1) from a primary carcinoma, Isreco2 (IS2) from a liver metastasis, and Isreco3 (IS3) from a peritoneal metastasis [27,73]. The cell lines were kindly provided by Richard Hamelin, INSERM, Paris, France. The normal colon samples from three patients with colorectal cancer were taken in a distance from the tumor sites. Microscopic evaluation of tissue sections stained by haematoxylin and eosin confirmed that the normal samples did not contain any tumor cells. For the primary carcinomas the median age at diagnosis was 75.5 years (range 58 – 88 years), and the median survival time for these patients was 116 months (range 13 – 147 months). The median age for patients with liver metastases was 71 years (range 55 – 75) with a median survival of 27 months (range 11 – 93). The median age for patients with carcinomatoses was 64.5 years (range 40 – 72) with a median survival at 28 months (range 19 – 65). The series consisted of 8 females and 18 males. Frozen sections were taken from all samples prior to RNA extraction, haematoxylin and eosin stained, and examined by a pathologist. All tumors were confirmed carcinomas and visually estimated to contain at least 40% tumor cells; for primaries the median was 70% (range: 40–90%) for liver metastases the median was 55% (range: 50–60%), and for the carcinomatoses 80% (range: 60–80%). The samples are taken from a research bio-bank registered at the National Health Institute and the project is approved by The Norwegian Data Inspectorate according to the national legislation.

### *TP53 *mutation status

DNA was extracted from tumor tissue pieces neighboring the ones used for RNA extraction (se below). All tumor samples were previously analyzed for *TP53 *mutations within exons 5–8 by screening for aberrantly migrating PCR fragments in constant denaturing gradient gel electrophoresis followed by identification of the specific mutations by direct sequencing (primary tumors, [31]; metastases, unpublished data).

### Total RNA extraction

The tissues were ground in liquid nitrogen and homogenized with a pellet pestle motor in 1ml of Trizol (Invitrogen, Carlsbad, CA). 0.2 ml of chloroform was added and the samples were vigorously shaken for 20s, and then incubated at RT for 5 min. After centrifugation at 12,000 × *g *for 15 min, the aqueous phase was mixed with 0.5 ml isopropanol. The RNA was allowed to precipitate for 10 min and collected after centrifugation at 12,000 × *g *for 10 min at 4°C. The RNA pellet was washed with 75% ethanol, collected after a brief centrifugation, air dried, and re-suspended in H_2_O at 55°C in 10 min. The purified RNA was quantified by spectrophotometer (NanoDrop 1000, NanoDrop Technologies, Boston, MA), and the quality was evaluated by capillary electrophoresis (Agilent 2100 Bioanalyzer, Agilent Technologies, Palo Alto, CA).

### Expression profiling

For each of the test and reference samples, 20 μg total RNA was reversely transcribed using the Agilent direct-label cDNA synthesis kit (Agilent Technologies) according to the manufacturer's directions. As a common reference for all samples, we used the "Universal Human Reference RNA", containing mRNA from ten cancer cell lines (Stratagene, La Jolla, CA). cDNA was labeled with cyanine 5-dCTP for test samples and cyanine 3-dCTP for the common reference (PerkinElmer Life Science, Boston, MA), and was purified using QIAquick PCR Purification columns (Qiagen, Valencia, CA). The cDNA was suspended in hybridization buffer and hybridized to Agilent Human 1A v2 22 k oligo microarrays (Agilent Technologies) for 17 h at 60°C according to the Agilent protocol. The slides were scanned by a laser confocal scanner (Agilent Technologies).

### Microarray data analyses

The image processing was performed with Agilent Feature Extraction 7.5 (Agilent Technologies). Local background subtraction and linear/LOWESS normalization were performed. Semi-processed values were imported into BASE (BioArray Software Environment; [74] customized for Agilent microarrays by the Norwegian Microarray Consortium), where spots with inadequate measurements were flagged and ratios calculated. Oligonucleotide probes with inadequate measurements in more than five of the 29 tumor samples were excluded from the analyses. For further analyses, we used data corresponding to 18 264 unique gene bank accession numbers, represented by 16 553 unique gene symbols [75].

BAMarray 2.0 (Bayesian ANOVA Analyses of Variation of Microarrays) [25] was used with default settings for detecting differentially expressed genes between two or more groups. BAMarray uses shrinkage estimation combined with model averaging. This provides a good balance between false rejection (the total number of genes falsely identified as being differentially expressed) and false non-rejections (the total number of genes falsely identified as being non-differentially expressed). By combing Z-cut and posterior variances from Bayesian ANOVA for microarray, we are likely to identify the differentially expressed target genes. Missing values were estimated in J-Express Pro 2.6 [76] with k-nearest neighbor imputation (k = 10). The most statistically significant genes associated with each group were reported with normal colon mucosa as the "baseline group".

Principal component analysis (PCA) and hierarchical cluster analysis (HCA) were performed in J-Express Pro 2.6 [76]. PCA reduces the dimensionality and detects structure in the relationships among variables (classify variables) [77]. HCA by use of average-linkage and Euclidean distance similarity measure was used to arrange variables according to groups based on their similarity. Afterwards, the results were visualized in a dendrogram. For each gene, expression values in tumor samples were centered over the median expression of the normal colon epithelial tissues before clustering.

### Quantitative real-time gene expression analyses

The mRNA expression of five potential target genes, *CCNE1*, *ELAC1*, *INCENP*, *PIAS2*, and *TM4SF1*, was measured by quantitative real-time fluorescence detection using TaqMan 7900 HT (Applied Biosystems, Foster City, CA). For each sample, cDNA was generated from five μg total RNA using a high capacity cDNA archive kit (Applied Biosystems) following the manufacturers' protocol. Ten ng cDNA was amplified for each gene using pre-designed assays (Hs00233356_m1, Hs00218846_m1, Hs00220336_m1, Hs00190699_m1, and Hs00371997_m1, respectively; Applied Biosystems). All samples were amplified in triplicates and the quantitative expression levels were measured against a standard curve generated from dilutions of cDNA from the human universal reference RNA (containing a mixture of RNA from ten different cell lines; Stratagene, CA). The median expression value of each sample was normalized against the average of the median of two endogenous controls, *ACTB *(4352935E; Applied Biosystems) and *GUSB *(4333767F; Applied Biosystems).

## Authors' contributions

KK carried the microarray experimental work, performed the statistical analyses, interpreted the results, and drafted the manuscript.

GEL performed RT-PCR experimental validation and participated in scientific discussions and manuscript preparation.

CBD participated in the statistical analyses and in the manuscript preparation.

LTB were responsible for the *TP53 *mutation analysis and participated in the study design.

GIM, JNM, TOR, KEG were responsible for referring the patients, collecting tissue specimens and for clinical information.

JNM re-examined all histological diagnoses and indicated representative tumor areas present in frozen sections taken from samples used for RNA extraction.

OM participated in scientific discussions and in the manuscript preparation.

RIS participated in statistical analysis, evaluation of data, and in the manuscript preparation.

RAL conceived the study, was responsible for its design and coordination and participated in evaluation of the data and in the manuscript preparation.

All authors have read and approved the final version of the manuscript.

## Supplementary Material

Additional file 1 List of 89 genes differentially expressed between primary and metastatic tumors. Click here for file

Additional file 2 List of 75 genes differentially expressed between tumors with or without mutated *TP53*. Click here for file

Additional file 3 Principal components and hierarchical clustering analyses of differentially expressed genes in colorectal carcinomas stratified by *TP53 *mutation status. A) Principal components analysis of 75 genes differentially expressed, assessed by BAMarray, in colorectal carcinomas stratified by *TP53 *mutation status. Red circles represent tumors with *TP53 *mutation, whereas black circles are wild type tumors. B) Dendrogram from hierarchical clustering analysis performed for the same genes (color-coding as in A). Click here for file

Additional file 4 Tumor clustering based on genes derived from cell lines modeling the metastasis process. Dendrogram from hierarchical clustering analysis of a panel of primary carcinomas (n = 18), liver metastases (n = 4), and carcinomatoses (n = 4), on genes associated with cell lines derived from tumors with different metastatic status. Click here for file

## References

[B1] The Cancer Registry of Norway. http://www.kreftregisteret.no.

[B2] Potter JD (1999). Colorectal cancer: molecules and populations. J Natl Cancer Inst.

[B3] Schena M, Shalon D, Davis RW, Brown PO (1995). Quantitative monitoring of gene expression patterns with a complementary DNA microarray. Science.

[B4] Zhang L, Zhou W, Velculescu VE, Kern SE, Hruban RH, Hamilton SR, Vogelstein B, Kinzler KW (1997). Gene expression profiles in normal and cancer cells. Science.

[B5] Perou CM, Sorlie T, Eisen MB, van de RM, Jeffrey SS, Rees CA, Pollack JR, Ross DT, Johnsen H, Akslen LA, Fluge O, Pergamenschikov A, Williams C, Zhu SX, Lonning PE, Borresen-Dale AL, Brown PO, Botstein D (2000). Molecular portraits of human breast tumors. Nature.

[B6] Bittner M, Meltzer P, Chen Y, Jiang Y, Seftor E, Hendrix M, Radmacher M, Simon R, Yakhini Z, Ben Dor A, Sampas N, Dougherty E, Wang E, Marincola F, Gooden C, Lueders J, Glatfelter A, Pollock P, Carpten J, Gillanders E (2000). Molecular classification of cutaneous malignant melanoma by gene expression profiling. Nature.

[B7] Chung CH, Parker JS, Karaca G, Wu J, Funkhouser WK, Moore D, Butterfoss D, Xiang D, Zanation A, Yin X, Shockley WW, Weissler MC, Dressler LG, Shores CG, Yarbrough WG, Perou CM (2004). Molecular classification of head and neck squamous cell carcinomas using patterns of gene expression. Cancer Cell.

[B8] Su AI, Welsh JB, Sapinoso LM, Kern SG, Dimitrov P, Lapp H, Schultz PG, Powell SM, Moskaluk CA, Frierson HF, Hampton GM (2001). Molecular classification of human carcinomas by use of gene expression signatures. Cancer Res.

[B9] 't Veer LJ, Dai H, van de Vijver MJ, He YD, Hart AA, Mao M, Peterse HL, van der KK, Marton MJ, Witteveen AT, Schreiber GJ, Kerkhoven RM, Roberts C, Linsley PS, Bernards R, Friend SH (2002). Gene expression profiling predicts clinical outcome of breast cancer. Nature.

[B10] Sorlie T, Perou CM, Tibshirani R, Aas T, Geisler S, Johnsen H, Hastie T, Eisen MB, van de RM, Jeffrey SS, Thorsen T, Quist H, Matese JC, Brown PO, Botstein D, Eystein LP, Borresen-Dale AL (2001). Gene expression patterns of breast carcinomas distinguish tumor subclasses with clinical implications. Proc Natl Acad Sci USA.

[B11] Ramaswamy S, Ross KN, Lander ES, Golub TR (2003). A molecular signature of metastasis in primary solid tumors. Nat Genet.

[B12] Beer DG, Kardia SL, Huang CC, Giordano TJ, Levin AM, Misek DE, Lin L, Chen G, Gharib TG, Thomas DG, Lizyness ML, Kuick R, Hayasaka S, Taylor JM, Iannettoni MD, Orringer MB, Hanash S (2002). Gene-expression profiles predict survival of patients with lung adenocarcinoma. Nat Med.

[B13] Glinsky GV, Glinskii AB, Stephenson AJ, Hoffman RM, Gerald WL (2004). Gene expression profiling predicts clinical outcome of prostate cancer. J Clin Invest.

[B14] Weiss MM, Kuipers EJ, Postma C, Snijders AM, Siccama I, Pinkel D, Westerga J, Meuwissen SG, Albertson DG, Meijer GA (2003). Genomic profiling of gastric cancer predicts lymph node status and survival. Oncogene.

[B15] Pomeroy SL, Tamayo P, Gaasenbeek M, Sturla LM, Angelo M, McLaughlin ME, Kim JY, Goumnerova LC, Black PM, Lau C, Allen JC, Zagzag D, Olson JM, Curran T, Wetmore C, Biegel JA, Poggio T, Mukherjee S, Rifkin R, Califano A (2002). Prediction of central nervous system embryonal tumor outcome based on gene expression. Nature.

[B16] Shih W, Chetty R, Tsao MS (2005). Expression profiling by microarrays in colorectal cancer (Review). Oncol Rep.

[B17] Buckhaults P, Rago C, St Croix B, Romans KE, Saha S, Zhang L, Vogelstein B, Kinzler KW (2001). Secreted and cell surface genes expressed in benign and malignant colorectal tumors. Cancer Res.

[B18] Kitahara O, Furukawa Y, Tanaka T, Kihara C, Ono K, Yanagawa R, Nita ME, Takagi T, Nakamura Y, Tsunoda T (2001). Alterations of gene expression during colorectal carcinogenesis revealed by cDNA microarrays after laser-capture microdissection of tumor tissues and normal epithelia. Cancer Res.

[B19] Agrawal D, Chen T, Irby R, Quackenbush J, Chambers AF, Szabo M, Cantor A, Coppola D, Yeatman TJ (2002). Osteopontin identified as lead marker of colon cancer progression, using pooled sample expression profiling. J Natl Cancer Inst.

[B20] Koehler A, Bataille F, Schmid C, Ruemmele P, Waldeck A, Blaszyk H, Hartmann A, Hofstaedter F, Dietmaier W (2004). Gene expression profiling of colorectal cancer and metastases divides tumors according to their clinicopathological stage. J Pathol.

[B21] Bertucci F, Salas S, Eysteries S, Nasser V, Finetti P, Ginestier C, Charafe-Jauffret E, Loriod B, Bachelart L, Montfort J, Victorero G, Viret F, Ollendorff V, Fert V, Giovaninni M, Delpero JR, Nguyen C, Viens P, Monges G, Birnbaum D (2004). Gene expression profiling of colon cancer by DNA microarrays and correlation with histoclinical parameters. Oncogene.

[B22] Bandres E, Catalan V, Sola I, Honorato B, Cubedo E, Cordeu L, Andion E, Escalada A, Zarate R, Salgado E, Zabalegui N, Garcia F, Garcia-Foncillas J (2004). Dysregulation of apoptosis is a major mechanism in the lymph node involvement in colorectal carcinoma. Oncol Rep.

[B23] Li M, Lin YM, Hasegawa S, Shimokawa T, Murata K, Kameyama M, Ishikawa O, Katagiri T, Tsunoda T, Nakamura Y, Furukawa Y (2004). Genes associated with liver metastasis of colon cancer, identified by genome-wide cDNA microarray. Int J Oncol.

[B24] D'Arrigo A, Belluco C, Ambrosi A, Digito M, Esposito G, Bertola A, Fabris M, Nofrate V, Mammano E, Leon A, Nitti D, Lise M (2005). Metastatic transcriptional pattern revealed by gene expression profiling in primary colorectal carcinoma. Int J Cancer.

[B25] Ishwaran H, Rao JS (2003). Detecting differentially expressed genes in microarrays using bayesian model selection. Journal of the American Statistical Association.

[B26] Diep CB, Teixeira MR, Thorstensen L, Wiig JN, Eknaes M, Nesland JM, Giercksky KE, Johansson B, Lothe RA (2004). Genome characteristics of primary carcinomas, local recurrences, carcinomatoses, and liver metastases from colorectal cancer patients. Mol Cancer.

[B27] Kleivi K, Teixeira MR, Eknaes M, Diep CB, Jakobsen KS, Hamelin R, Lothe RA (2004). Genome signatures of colon carcinoma cell lines. Cancer Genet Cytogenet.

[B28] Saha S, Bardelli A, Buckhaults P, Velculescu VE, Rago C, St Croix B, Romans KE, Choti MA, Lengauer C, Kinzler KW, Vogelstein B (2001). A phosphatase associated with metastasis of colorectal cancer. Science.

[B29] Yanagawa R, Furukawa Y, Tsunoda T, Kitahara O, Kameyama M, Murata K, Ishikawa O, Nakamura Y (2001). Genome-wide screening of genes showing altered expression in liver metastases of human colorectal cancers by cDNA microarray. Neoplasia.

[B30] Jarry A, Vallette G, Cassagnau E, Moreau A, Bou-Hanna C, Lemarre P, Letessier E, Le Neel JC, Galmiche JP, Laboisse CL (1999). Interleukin 1 and interleukin 1beta converting enzyme (caspase 1) expression in the human colonic epithelial barrier. Caspase 1 downregulation in colon cancer. Gut.

[B31] Diep CB, Thorstensen L, Meling GI, Skovlund E, Rognum TO, Lothe RA (2003). Genetic tumor markers with prognostic impact in dukes' stages B and C colorectal cancer patients. J Clin Oncol.

[B32] Yanaihara N, Kohno T, Takakura S, Takei K, Otsuka A, Sunaga N, Takahashi M, Yamazaki M, Tashiro H, Fukuzumi Y, Fujimori Y, Hagiwara K, Tanaka T, Yokota J (2001). Physical and transcriptional map of a 311-kb segment of chromosome 18q21, a candidate lung tumor suppressor locus. Genomics.

[B33] Lampson MA, Renduchitala K, Khodjakov A, Kapoor TM (2004). Correcting improper chromosome-spindle attachments during cell division. Nat Cell Biol.

[B34] Praml C, Amler LC, Dihlmann S, Finke LH, Schlag P, Schwab M (1998). Secretory type II phospholipase A2 (PLA2G2A) expression status in colorectal carcinoma derived cell lines and in normal colonic mucosa. Oncogene.

[B35] Marken JS, Schieven GL, Hellstrom I, Hellstrom KE, Aruffo A (1992). Cloning and expression of the tumor-associated antigen L6. Proc Natl Acad Sci USA.

[B36] Kao YR, Shih JY, Wen WC, Ko YP, Chen BM, Chan YL, Chu YW, Yang PC, Wu CW, Roffler SR (2003). Tumor-associated antigen L6 and the invasion of human lung cancer cells. Clin Cancer Res.

[B37] Prescott JE, Osthus RC, Lee LA, Lewis BC, Shim H, Barrett JF, Guo Q, Hawkins AL, Griffin CA, Dang CV (2001). A novel c-Myc-responsive gene, JPO1, participates in neoplastic transformation. J Biol Chem.

[B38] Notterman DA, Alon U, Sierk AJ, Levine AJ (2001). Transcriptional gene expression profiles of colorectal adenoma, adenocarcinoma, and normal tissue examined by oligonucleotide arrays. Cancer Res.

[B39] Zou TT, Selaru FM, Xu Y, Shustova V, Yin J, Mori Y, Shibata D, Sato F, Wang S, Olaru A, Deacu E, Liu TC, Abraham JM, Meltzer SJ (2002). Application of cDNA microarrays to generate a molecular taxonomy capable of distinguishing between colon cancer and normal colon. Oncogene.

[B40] Williams NS, Gaynor RB, Scoggin S, Verma U, Gokaslan T, Simmang C, Fleming J, Tavana D, Frenkel E, Becerra C (2003). Identification and validation of genes involved in the pathogenesis of colorectal cancer using cDNA microarrays and RNA interference. Clin Cancer Res.

[B41] Li A, Varney ML, Singh RK (2004). Constitutive expression of growth regulated oncogene (gro) in human colon carcinoma cells with different metastatic potential and its role in regulating their metastatic phenotype. Clin Exp Metastasis.

[B42] Birkenkamp-Demtroder K, Olesen SH, Sorensen FB, Laurberg S, Laiho P, Aaltonen LA, ORntoft TF (2005). Differential gene expression in colon cancer of the caecum versus the sigmoid and rectosigmoid. Gut.

[B43] Yang SK, Choi MS, Kim OH, Myung SJ, Jung HY, Hong WS, Kim JH, Min YI (2002). The increased expression of an array of C-X-C and C-C chemokines in the colonic mucosa of patients with ulcerative colitis: regulation by corticosteroids. Am J Gastroenterol.

[B44] Friedl A, Stoesz SP, Buckley P, Gould MN (1999). Neutrophil gelatinase-associated lipocalin in normal and neoplastic human tissues. Cell type-specific pattern of expression. Histochem J.

[B45] Zucker S, Vacirca J (2004). Role of matrix metalloproteinases (MMPs) in colorectal cancer. Cancer Metastasis Rev.

[B46] Croner RS, Foertsch T, Brueckl WM, Guenther K, Siebenhaar R, Stremmel C, Matzel KE, Papadopoulos T, Kirchner T, Behrens J, Klein-Hitpass L, Stuerzl M, Hohenberger W, Reingruber B (2005). Common denominator genes that distinguish colorectal carcinoma from normal mucosa. Int J Colorectal Dis.

[B47] Moss SF, Krivosheyev V, de Souza A, Chin K, Gaetz HP, Chaudhary N, Worman HJ, Holt PR (1999). Decreased and aberrant nuclear lamin expression in gastrointestinal tract neoplasms. Gut.

[B48] Clark ME, Kelner GS, Turbeville LA, Boyer A, Arden KC, Maki RA (2000). ADAMTS9, a novel member of the ADAM-TS/metallospondin gene family. Genomics.

[B49] Porter S, Clark IM, Kevorkian L, Edwards DR (2005). The ADAMTS metalloproteinases. Biochem J.

[B50] Lind GE, Kleivi K, Meling GI, Teixeira MR, Thiis-Evensen E, Rognum T, Lothe RA (2006). *ADAMTS1*, *CRABP1*, and *NR3C1 *identified as epigenetically deregulated genes in colorectal tumorigenesis. Cell Oncol.

[B51] Thompson J, Seitz M, Chastre E, Ditter M, Aldrian C, Gespach C, Zimmermann W (1997). Down-regulation of carcinoembryonic antigen family member 2 expression is an early event in colorectal tumorigenesis. Cancer Res.

[B52] Wanzel M, Kleine-Kohlbrecher D, Herold S, Hock A, Berns K, Park J, Hemmings B, Eilers M (2005). Akt and 14-3-3eta regulate Miz1 to control cell-cycle arrest after DNA damage. Nat Cell Biol.

[B53] Wu S, Cetinkaya C, Munoz-Alonso MJ, von der LN, Bahram F, Beuger V, Eilers M, Leon J, Larsson LG (2003). Myc represses differentiation-induced p21CIP1 expression via Miz-1-dependent interaction with the p21 core promoter. Oncogene.

[B54] Schmidt D, Muller S (2002). Members of the PIAS family act as SUMO ligases for c-Jun and p53 and repress p53 activity. Proc Natl Acad Sci USA.

[B55] Komori T, Takemasa I, Higuchi H, Yamasaki M, Ikeda M, Yamamoto H, Ohue M, Nakamori S, Sekimoto M, Matsubara K, Monden M (2004). Identification of differentially expressed genes involved in colorectal carcinogenesis using a cDNA microarray. J Exp Clin Cancer Res.

[B56] Mori Y, Selaru FM, Sato F, Yin J, Simms LA, Xu Y, Olaru A, Deacu E, Wang S, Taylor JM, Young J, Leggett B, Jass JR, Abraham JM, Shibata D, Meltzer SJ (2003). The impact of microsatellite instability on the molecular phenotype of colorectal tumors. Cancer Res.

[B57] Dunican DS, McWilliam P, Tighe O, Parle-McDermott A, Croke DT (2002). Gene expression differences between the microsatellite instability (MIN) and chromosomal instability (CIN) phenotypes in colorectal cancer revealed by high-density cDNA array hybridization. Oncogene.

[B58] Zeng ZJ, Yang LY, Ding X, Wang W (2004). Expressions of cysteine-rich61, connective tissue growth factor and Nov genes in hepatocellular carcinoma and their clinical significance. World J Gastroenterol.

[B59] Shimizu T, Okayama A, Inoue T, Takeda K (2005). Analysis of gene expression during staurosporine-induced neuronal differentiation of human prostate cancer cells. Oncol Rep.

[B60] Jiang WG, Watkins G, Fodstad O, Douglas-Jones A, Mokbel K, Mansel RE (2004). Differential expression of the CCN family members Cyr61, CTGF and Nov in human breast cancer. Endocr Relat Cancer.

[B61] Chang CC, Shih JY, Jeng YM, Su JL, Lin BZ, Chen ST, Chau YP, Yang PC, Kuo ML (2004). Connective tissue growth factor and its role in lung adenocarcinoma invasion and metastasis. J Natl Cancer Inst.

[B62] Lin BR, Chang CC, Che TF, Chen ST, Chen RJ, Yang CY, Jeng YM, Liang JT, Lee PH, Chang KJ, Chau YP, Kuo ML (2005). Connective tissue growth factor inhibits metastasis and acts as an independent prognostic marker in colorectal cancer. Gastroenterology.

[B63] Bieche I, Tozlu S, Girault I, Lidereau R (2004). Identification of a three-gene expression signature of poor-prognosis breast carcinoma. Mol Cancer.

[B64] Miller CT, Moy JR, Lin L, Schipper M, Normolle D, Brenner DE, Iannettoni MD, Orringer MB, Beer DG (2003). Gene amplification in esophageal adenocarcinomas and Barrett's with high-grade dysplasia. Clin Cancer Res.

[B65] Yasui K, Arii S, Zhao C, Imoto I, Ueda M, Nagai H, Emi M, Inazawa J (2002). TFDP1, CUL4A, and CDC16 identified as targets for amplification at 13q34 in hepatocellular carcinomas. Hepatology.

[B66] Li JQ, Miki H, Ohmori M, Wu F, Funamoto Y (2001). Expression of cyclin E and cyclin-dependent kinase 2 correlates with metastasis and prognosis in colorectal carcinoma. Hum Pathol.

[B67] Bardi G, Pandis N, Fenger C, Kronborg O, Bomme L, Heim S (1993). Deletion of 1p36 as a primary chromosomal aberration in intestinal tumorigenesis. Cancer Res.

[B68] Nishimoto T, Seino H, Seki N, Hori TA (1994). The human CHC1 gene encoding RCC1 (regulator of chromosome condensation) (CHC1) is localized to human chromosome 1p36.1. Genomics.

[B69] Fearon ER, Vogelstein B (1990). A genetic model for colorectal tumorigenesis. Cell.

[B70] Sørlie T (2004). Molecular portraits of breast cancer: tumor subtypes as distinct disease entities. Eur J Cancer.

[B71] Alon U, Barkai N, Notterman DA, Gish K, Ybarra S, Mack D, Levine AJ (1999). Broad patterns of gene expression revealed by clustering analysis of tumor and normal colon tissues probed by oligonucleotide arrays. Proc Natl Acad Sci USA.

[B72] Ross DT, Scherf U, Eisen MB, Perou CM, Rees C, Spellman P, Iyer V, Jeffrey SS, van de RM, Waltham M, Pergamenschikov A, Lee JC, Lashkari D, Shalon D, Myers TG, Weinstein JN, Botstein D, Brown PO (2000). Systematic variation in gene expression patterns in human cancer cell lines. Nat Genet.

[B73] Cajot JF, Sordat I, Silvestre T, Sordat B (1997). Differential display cloning identifies motility-related protein (MRP1/CD9) as highly expressed in primary compared to metastatic human colon carcinoma cells. Cancer Res.

[B74] Saal LH, Troein C, Vallon-Christersson J, Gruvberger S, Borg A, Peterson C (2002). BioArray Software Environment (BASE): a platform for comprehensive management and analysis of microarray data. Genome Biol.

[B75] TIGR resourerer April 2005. Chromosome locations and cytobands extracted from Source May 2005.

[B76] Dysvik B, Jonassen I (2001). J-Express: exploring gene expression data using Java. Bioinformatics.

[B77] Raychaudhuri S, Stuart JM, Altman RB (2002). Principal component analysis to summarize microarray experiments: application to sporulation time series. Pac Symp Biocomput.

