# Supplementary material for: Gene expression profiles of primary colorectal carcinomas, liver metastases, and carcinomatoses
Source: Mol Cancer. 2007 Jan 3;6:2. doi: 10.1186/1476-4598-6-2 (PMC1770935; doi:10.1186/1476-4598-6-2)
Supplement: Additional file 1 — List of 89 genes differentially expressed between primary and metastatic tumors. [file 1476-4598-6-2-S1.pdf]

Additional file 1. Eighty-nine genes differentially expressed between primary and metastatic tumors.

GenBank acc	Gene symbol	Gene name	Z-cut	Stage	Fold change liver	Fold change carcinomatoses	Fold change primary
AB029151	*ELAC1*	elaC homolog 1 (E. coli)	-4,41	M	-2.56	-2.89	-1.37
AB037848	*SYT13*	synaptotagmin XIII	-4,21	M	-11.18	-3.87	-1.5
AF183428	*MRPL16*	mitochondrial ribosomal protein L16	-4,05	M	-2.81	-2.67	-1.46
BC009707	*MRPL40*	mitochondrial ribosomal protein L40	-3,97	M	-2.04	-2.13	-1.15
AL160131	*C22orf18*	chromosome 22 open reading frame 18	-3,70	M	-2.5	-2.26	-1.59
NM_025079	*FLJ23231*	hypothetical protein FLJ23231	-3,42	M	-3.44	-2.42	-1.1
NM_012145	*DTYMK*	deoxythymidylate kinase (thymidylate kinase)	-3,39	M	-3.04	-2.33	-1.37
M81057	*CPB1*	carboxypeptidase B1 (tissue)	3,38	M	6.72	2.64	2.28
NM_005739	*RASGRP1*	RAS guanyl releasing protein 1 (calcium and DAG-regulated)	-3,37	M	-2.45	-2.25	-1.15
NM_017590	*RoXaN*	rotavirus X protein associated with NSP3	-3,35	M	-2.41	-3.04	-1.32
BC004398	*MGC20533*	similar to RIKEN cDNA 2410004L22 gene (M. musculus)	-3,33	M	-2.45	-2.12	-1.36
NM_152288	*MGC13024*	hypothetical protein MGC13024	3,30	M	7.58	5.28	2.01
BC008607	*TIMM13*	translocase of inner mitochondrial membrane 13 homolog (yeast)	-3,30	M	-2.12	-2.12	-1.32
NM_023088	*CAPN10*	calpain 10	-3,30	M	-2	-2.07	-1.07
AK097373	*CYP4Z2P*	cytochrome P450 4Z2 pseudogene	3,21	M	6.36	2.51	2.05
BC009244	*IDH2*	isocitrate dehydrogenase 2 (NADP+), mitochondrial	-3,17	M	-2.99	-2.38	-1.38
S94541			-3,16	M	-2.95	-2.1	-1.23
NM_024805	*C18orf22*	chromosome 18 open	-3,13	M	-2.54	-3.12	-1.6

		reading frame 22					
X98311	*CEACAM7*	carcinoembryonic antigen-related cell adhesion molecule 7	3,13	M	5.33	2.44	1.93
NM_015492	*DKFZP434H132*	DKFZP434H132 protein	-3,09	M	-2.6	-2.42	-1.73
BC033068	*PCDHB13*	protocadherin beta 13	3,07	M	2.32	2.6	1.77
NM_001406	*EFNB3*	ephrin-B3	3,06	M	2.71	2.37	1.58
M87507	*CASP1*	caspase 1, apoptosis-related cysteine protease (interleukin 1, beta, convertase)	-3,04	M	-3.21	-3.18	-1.42
AK096323	*SGPP2*	sphingosine-1-phosphate phosphotase 2	-3,03	M	-6.25	-2.95	-1.53
AK056195	*INCENP*	inner centromere protein antigens 135/155kDa	-3,00	M	-2.15	-2.56	-1.54
AJ012590	*H6PD*	hexose-6-phosphate dehydrogenase (glucose 1-dehydrogenase)	2,98	M	2.87	2.93	1.88
NM_176888	*TAS2R48*	taste receptor, type 2, member 48	2,98	M	3.9	3.82	1.6
M22430	*PLA2G2A*	phospholipase A2, group IIA (platelets, synovial fluid)	-2,97	M	-25.03	-17.22	-2.29
AF328788	*AMN*	amnionless homolog (mouse)	2,96	M	3.24	2.47	1.66
X87342	*LLGL2*	lethal giant larvae homolog 2 (Drosophila)	2,95	M	2.85	3.35	1.92
AF227137	*TAS2R13*		2,93	M	5.71	2.21	1.71
AB022718	*C10orf10*	chromosome 10 open reading frame 10	2,93	M	2.76	2.25	1.65
BC034145	*TM4SF1*	transmembrane 4 superfamily member 1	2,92	M	3.22	4.76	1.45
AF081535	*CDC45L*	CDC45 cell division cycle 45-like (S. cerevisiae)	-2,92	M	-2.53	-2.04	-1.35
NM_012226	*P2RX2*	purinergic receptor P2X, ligand-gated ion channel, 2	2,90	M	2.87	2.07	1.68
NM_080612	*GAB3*	GRB2-associated binding protein 3	-2,90	M	-2.64	-2.24	-1.13

BC012125	*SLC39A8*	solute carrier family 39 (zinc transporter), member 8	-2,86	M	-3.17	-2.14	-1.26
BC029055	*PGA5*	pepsinogen 5, group I (pepsinogen A)	2,86	M	2.41	2.03	1.5
D13639	*CCND2*	cyclin D2	-2,85	M	-4.17	-2.26	1.2
NM_031213	*C19orf27*	chromosome 19 open reading frame 27	2,84	M	2.18	2.07	1.45
BC016308	*CXCL3*	chemokine (C-X-C motif) ligand 3	3,77	P	1.88	1.83	5.96
M98398	*CD36*	CD36 antigen (collagen type I receptor, thrombospondin receptor)	-3,15	P	-3.39	-1.78	-3.64
NM_017899	*TSC*	hypothetical protein FLJ20607	3,11	P	2.32	1.62	3.76
NM_032865	*CTEN*	C-terminal tensin-like	3,05	P	3.74	1.59	3.58
NM_025113	*C13orf18*	chromosome 13 open reading frame 18	3,02	P	1.87	1.96	2.81
NM_001036	*RYR3*	ryanodine receptor 3	-2,97	P	-1.73	-2.17	-2.81
NM_033046	*RTKN*	rhotekin	2,96	P	1.5	1.27	2.03
NM_017870	*HSPA5BP1*	heat shock 70kDa protein 5 (glucose-regulated protein, 78kDa) binding protein 1	2,95	P	1.37	1.14	2.24
NM_018192	*LEPREL1*	leprecan-like 1	-2,94	P	-1.56	-1.36	-2.38
X52486	*UNG2*	uracil-DNA glycosylase 2	2,93	P	2	1.33	3.19
D42073	*RCN1*	reticulocalbin 1, EF-hand calcium binding domain	2,90	P	-1.04	1.32	2
NM_003226	*TFF3*	trefoil factor 3 (intestinal)	-2,89	P	-2.99	1.27	-2.42
L15203	*TFF3*	trefoil factor 3 (intestinal)	-2,88	P	-2.91	1.37	-2.74
BC011976	*CXCL1*	chemokine (C-X-C motif) ligand 1 (melanoma growth stimulating activity, alpha)	2,83	P	-1.27	-1.32	3.6
NM_024889	*C10orf81*	chromosome 10 open reading frame 81	2,82	P	-1.21	1.35	2.64
BC020791	*TTR*	transthyretin (prealbumin, amyloidosis type I)	-2,82	P	-1.27	-2.63	-2.9
D83548	*LIPC*	lipase, hepatic	-2,81	P	-1.43	-2.21	-3.27

NM_014759	*PHYHIP*	phytanoyl-CoA hydroxylase interacting protein	-2,79	P	-1.95	-2.32	-2.13
J02984	*RPS15*	ribosomal protein S15	2,77	P	1.68	1.95	2.43
NM_003734	*AOC3*	amine oxidase, copper containing 3 (vascular adhesion protein 1)	-2,77	P	-1.79	-1.43	-2.41
AF480461	*ZAK*	sterile alpha motif and leucine zipper containing kinase AZK	2,74	P	1.31	1.42	2.31
BC028600	*SLC20A2*	solute carrier family 20 (phosphate transporter), member 2	-2,69	P	-1.23	-1.83	-2.06
AK056089	*AF1Q*	ALL1-fused gene from chromosome 1q	-2,67	P	-2.56	-2.25	-2.83
NM_020685	*C3orf14*	chromosome 3 open reading frame 14	-2,67	P	-1.87	-1.26	-2.31
U37100	*AKR1B10*	aldo-keto reductase family 1, member B10 (aldose reductase)	-2,64	P	-2.98	-2.23	-3.25
AB038518	*COLEC12*	collectin sub-family member 12	-2,62	P	-3.34	-2.37	-3.6
BC022012	*VSNL1*	visinin-like 1	2,60	P	1.01	1.41	2.08
BC033089	*LCN2*	lipocalin 2 (oncogene 24p3)	2,59	P	1.56	7.08	11.2
BC039733	*PTX3*	pentaxin-related gene, rapidly induced by IL-1 beta	-2,58	P	-2.02	-1.62	-2.57
NM_080284	*ABCA6*	ATP-binding cassette, sub-family A (ABC1), member 6	-2,57	P	-2.2	-2.07	-2.81
NM_152552	*SAMD3*	sterile alpha motif domain containing 3	2,55	P	2.68	2.44	2.38
S65738	*DSTN*	destrin (actin depolymerizing factor)	-2,54	P	-2.78	-1.65	-2.64
BC002780	*FMO4*	flavin containing monooxygenase 4	-2,51	P	-1.5	-1.88	-2.18
BC022250	*FLJ14054*	hypothetical protein FLJ14054	-2,49	P	-2.16	1.18	-3.47
NM_002089	*CXCL2*	chemokine (C-X-C motif)	2,48	P	-1.26	-1.84	2.61

		ligand 2					
U10550	*GEM*	GTP binding protein overexpressed in skeletal muscle	-2,48	P	-2.71	-1.36	-2.83
AJ001014	*RAMP1*	receptor (calcitonin) activity modifying protein 1	-2,46	P	-1.65	-2.04	-2.29
NM_023926	*FLJ12895*	hypothetical protein FLJ12895	-2,46	P	-4.72	-1.45	-2.8
NM_005916	*MCM7*	MCM7 minichromosome maintenance deficient 7 (S. cerevisiae)	2,45	P	1.81	1.86	2.13
BC012295	*LMNB1*	lamin B1	-2,45	P	-1.9	-1.89	-2.28
NM_023005	*BAZ1B*	bromodomain adjacent to zinc finger domain, 1B	2,44	P	1.19	1.52	2.03
AK023453	*FLJ13391*	hypothetical protein FLJ13391	2,43	P	1.67	2.24	2.48
BC040071	*A2M*	alpha-2-macroglobulin	-2,42	P	-5.12	-1.64	-3.48
BC040288	*FLJ23342*	hypothetical protein FLJ23342	2,41	P	1.28	1.07	2.36
BC013575	*PLAU*	plasminogen activator, urokinase	2,40	P	1.31	2.82	3.04
NM_016397	*TH1L*	TH1-like (Drosophila)	2,39	P	3.16	1.36	2.39
NM_022972	*FGFR2*	fibroblast growth factor receptor 2	-2,37	P	-2.2	-1.65	-2.53
NM_020161	*DKFZp547H025*	hypothetical protein DKFZp547H025	-2,34	P	-1.31	-1.62	-2
NM_031942	*CDCA7*	cell division cycle associated 7	2,32	P	1.08	2.04	3.24

Z-cut is derived from BAM. M; metastases from liver and peritoneum, P; primary carcinomas, L; liver metastases, C; carcinomatoses. Fold change; expression in fold change using medians of each group as compared to normal colonic tissue.
