# Supplementary material for: Gene expression profiles of primary colorectal carcinomas, liver metastases, and carcinomatoses
Source: Mol Cancer. 2007 Jan 3;6:2. doi: 10.1186/1476-4598-6-2 (PMC1770935; doi:10.1186/1476-4598-6-2)
Supplement: Additional file 2 — List of 75 genes differentially expressed between tumors with or without mutated TP53. [file 1476-4598-6-2-S2.pdf]

Additional file 2. Seventy-five genes differentially expressed between tumors with or without mutated *TP53*.

GenBank Acc	*Gene Symbol*	*Gene Name*	*TP53 status*	*Z-cut*	*Fold change TP53 mut*	*Fold change TP53 wt*
NM_153366	*SVEP1*	sushi, von Willebrand factor type A, EGF and pentraxin domain containing 1	mut	-2,42	-2.67	-1.88
M60459	*EPOR*	erythropoietin receptor	mut	-2,43	-2.13	-1.27
AL050262	*TLR1*	toll-like receptor 1	mut	-2,44	-3.45	-2.05
X14362	*CR1*	complement component (3b/4b) receptor 1, including Knops blood group system	mut	-2,44	-2.45	-1.93
NM_014912	*CPEB3*	cytoplasmic polyadenylation element binding protein 3	mut	-2,47	-2.04	-1.31
AF218012	*FER1L4*	fer-1-like 4 (C. elegans)	mut	2,49	2.84	-1.12
D87845	*PAFAH2*	platelet-activating factor acetylhydrolase 2, 40kDa	mut	2,49	3.49	1.76
AB067469	*T1*	Tularik gene 1	mut	2,49	3.50	2.39
NM_019043	*APBB1IP*	amyloid beta (A4) precursor protein-binding, family B, member 1 interacting protein	mut	-2,50	-3.07	-1.60
NM_031474	*NRIP2*	nuclear receptor interacting protein 2	mut	2,51	2.07	1.38
AL049940	*RYBP*	RING1 and YY1 binding protein	mut	-2,52	-2.19	-1.47
AF124733	*IRX4*	iroquois homeobox protein 4	mut	2,53	2.25	1.78
NM_144712	*SLC23A3*	solute carrier family 23 (nucleobase transporters), member 3	mut	2,58	2.04	1.64
BC023312	*SIAT10*	sialyltransferase 10 (alpha-2,3-sialyltransferase VI)	mut	-2,60	-2.26	-1.45
NM_025034	*USH1C*	Usher syndrome 1C (autosomal recessive, severe)	mut	2,60	2.11	1.43
BC036543	*NMUR1*	neuromedin U receptor 1	mut	2,60	2.38	1.43
NM_031426	*C9orf58*	chromosome 9 open reading frame 58	mut	-2,69	-3.48	-2.21
AK057903	*SLC31A2*	solute carrier family 31 (copper transporters), member 2	mut	-2,69	-3.83	-1.80
NM_017895	*DDX27*	DEAD (Asp-Glu-Ala-Asp) box polypeptide 27	mut	2,70	2.06	1.22
NM_080616	*C20orf112*	chromosome 20 open reading frame 112	mut	2,73	2.02	1.39
BC031307	*RPS27L*	ribosomal protein S27-like	mut	-2,75	-2.26	-1.35
NM_004001	*FCGR2B*	Fc fragment of IgG, low affinity IIb, receptor for (CD32)	mut	-2,77	-3.74	-1.60
BC015511	*IL6*	interleukin 6 (interferon, beta 2)	mut	-2,79	-3.63	-1.41

NM_003106	*SOX2*	SRY (sex determining region Y)-box 2	mut	-2,79	-2.39	-1.70
AK096919	*ADCK1*	aarF domain containing kinase 1	mut	-2,81	-2.02	-1.54
AB018549	*LY96*	lymphocyte antigen 96	mut	-2,93	-2.12	-1.58
NM_032664	*FUT10*	fucosyltransferase 10 (alpha (1,3) fucosyltransferase)	mut	-2,97	-2.10	-1.31
NM_003716	*CADPS*	Ca2+-dependent secretion activator	mut	3,08	3.96	1.43
U31202	*NOG*	noggin	mut	-3,14	-2.59	-1.53
AF152462	*ITM2B*	integral membrane protein 2B	mut	-3,16	-2.41	-1.49
NM_015833	*ADARB1*	adenosine deaminase, RNA-specific, B1 (RED1 homolog rat)	mut	-3,22	-2.10	-1.32
BC005902	*BLVRA*	biliverdin reductase A	mut	-3,28	-2.47	-1.38
NM_024693	*ECHDC3*	enoyl Coenzyme A hydratase domain containing 3	mut	-3,49	-4.02	-1.21
NM_007127	*VIL1*	villin 1	wt	-2,24	-1.44	-2.01
NM_138799	*OACT2*	O-acyltransferase (membrane bound) domain containing 2	wt	-2,24	-2.19	-2.41
J04813	*CYP3A5*	cytochrome P450, family 3, subfamily A, polypeptide 5	wt	-2,26	-1.48	-3.10
NM_133476	*ZNF384*	zinc finger protein 384	wt	2,26	-1.42	2.06
BC002666	*GBP1*	guanylate binding protein 1, interferon-inducible, 67kDa	wt	2,28	-1.04	2.43
AK098106	*LOC120224*	hypothetical protein BC016153	wt	-2,30	-1.97	-2.02
BC015842	*EIF4A2*	eukaryotic translation initiation factor 4A, isoform 2	wt	2,30	1.64	2.24
AK075372	*SFRP2*	secreted frizzled-related protein 2	wt	2,31	1.96	2.32
NM_012132			wt	-2,31	-2.25	-2.54
AK095944	*PIB5PA*	phosphatidylinositol (4,5) bisphosphate 5-phosphatase, A	wt	-2,33	-1.73	-3.23
NM_019062	*FLJ20225*	hypothetical protein FLJ20225	wt	-2,33	-1.30	-3.02
AF237813	*ABAT*	4-aminobutyrate aminotransferase	wt	-2,34	-1.93	-2.93
M60028	*HLA-DQB1*	major histocompatibility complex, class II, DQ beta 1	wt	2,34	1.55	2.19
NM_032720	*SPON1*	spondin 1, extracellular matrix protein	wt	2,34	1.72	2.30
NM_005495	*SLC17A4*	solute carrier family 17 (sodium phosphate), member 4	wt	-2,37	-1.03	-3.01
X07315	*NUTF2*	nuclear transport factor 2	wt	2,39	1.83	2.05
NM_002591	*PCK1*	phosphoenolpyruvate carboxykinase 1 (soluble)	wt	-2,39	-2.38	-4.94

BC008182	*DNAJA1*	DnaJ (Hsp40) homolog, subfamily A, member 1	wt	2,39	1.63	2.15
AB044547	*HNRPL*	heterogeneous nuclear ribonucleoprotein L	wt	2,40	1.06	2.56
AK024667	*PCTP*	phosphatidylcholine transfer protein	wt	-2,40	-1.47	-2.07
BC004350	*BAT1*	HLA-B associated transcript 1	wt	2,42	2.62	3.41
AF007170	*C1orf34*	chromosome 1 open reading frame 34	wt	-2,44	-2.06	-2.58
NM_138480	*PLEKHA6*	pleckstrin homology domain containing, family A member 6	wt	-2,45	-1.67	-2.51
NM_017678	*C11orf33*	chromosome 11 open reading frame 33	wt	-2,46	-2.33	-7.56
AF048700	*SPINK4*	serine protease inhibitor, Kazal type 4	wt	-2,51	-1.73	-20.33
NM_015973	*GAL*	galanin	wt	-2,52	-1.29	-2.65
NM_152597	*FSIP1*	fibrous sheath interacting protein 1	wt	-2,54	-2.51	-3.09
BC028600	*SLC20A2*	solute carrier family 20 (phosphate transporter), member 2	wt	-2,60	-1.67	-2.07
BC020791	*TTR*	transthyretin (prealbumin, amyloidosis type I)	wt	-2,65	-2.73	-2.68
NM_003740	*KCNK5*	potassium channel, subfamily K, member 5	wt	-2,65	-1.79	-3.17
M30640	*SELE*	selectin E (endothelial adhesion molecule 1)	wt	-2,66	-1.57	-2.81
D49400	*ATP6V1F*	ATPase, H+ transporting, lysosomal 14kDa, V1 subunit F	wt	2,69	1.47	2.09
D78367	*KRT12*	keratin 12 (Meesmann corneal dystrophy)	wt	-2,73	-2.17	-2.99
BC005278	*GCG*	glucagon	wt	-2,77	-6.32	-10.30
U37100	*AKR1B10*	aldo-keto reductase family 1, member B10 (aldose reductase)	wt	-2,81	-2.38	-3.32
AK024899	*ENPP3*	ectonucleotide pyrophosphatase/phosphodiesterase 3	wt	-2,81	-1.60	-3.96
M61857	*CYP2C9*	cytochrome P450, family 2, subfamily C, polypeptide 9	wt	-2,88	-3.43	-4.64
NM_017542	*POGK*	pogo transposable element with KRAB domain	wt	2,89	1.77	2.51
AF042832	*FOXD2*	forkhead box D2	wt	-3,05	-1.42	-2.37
AK093330	*IGFL2*	insulin growth factor-like family member 2	wt	3,14	1.34	3.11
BC025671	*SLC26A3*	solute carrier family 26, member 3	wt	-3,27	-4.79	-28.27
BC009698	*APOC1*	apolipoprotein C-I	wt	3,64	1.27	4.01

Mut; mutated, wt; wild type. Z-cut is derived from BAM. Fold change; expression in fold change using medians of each group as compared to normal colonic tissue.
