# Supplementary figures and images for: Gene expression profiles of primary colorectal carcinomas, liver metastases, and carcinomatoses
Source: Mol Cancer. 2007 Jan 3;6:2. doi: 10.1186/1476-4598-6-2 (PMC1770935; doi:10.1186/1476-4598-6-2)

A)

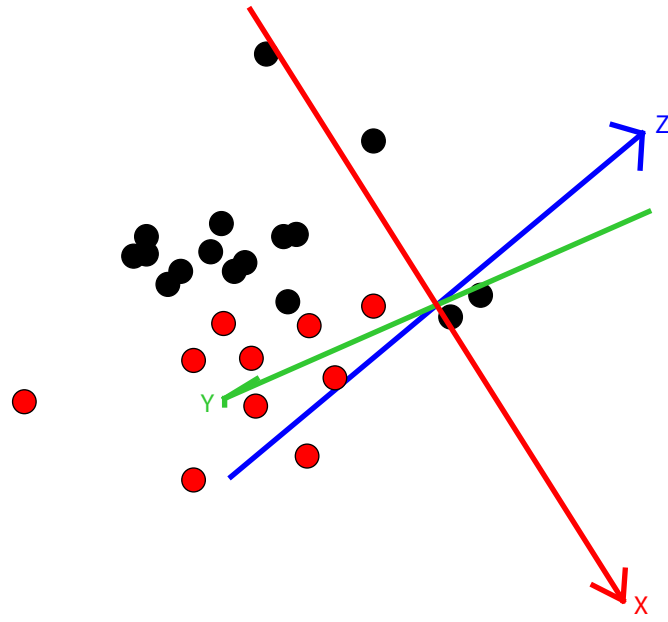

B)

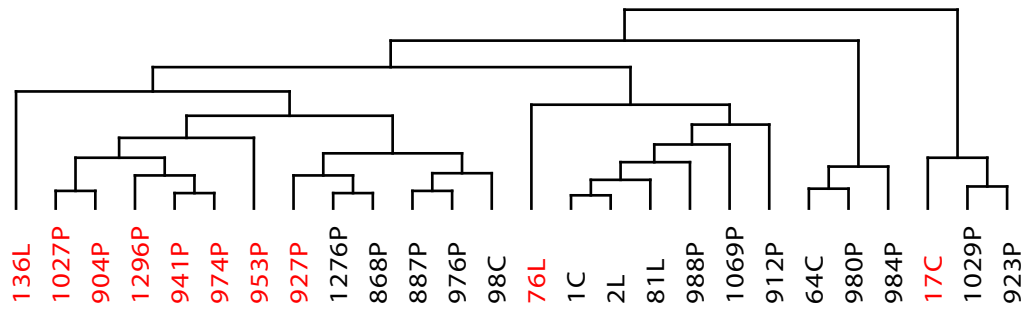

Supplement: Additional file 3 — Principal components and hierarchical clustering analyses of differentially expressed genes in colorectal carcinomas stratified by TP53 mutation status. A) Principal components analysis of 75 genes differentially expressed, assessed by BAMarray, in colorectal carcinomas stratified by TP53 mutation status. Red circles represent tumors with TP53 mutation, whereas black circles are wild type tumors. B) Dendrogram from hierarchical clustering analysis performed for the same genes (color-coding as in A). [file 1476-4598-6-2-S3.pdf]

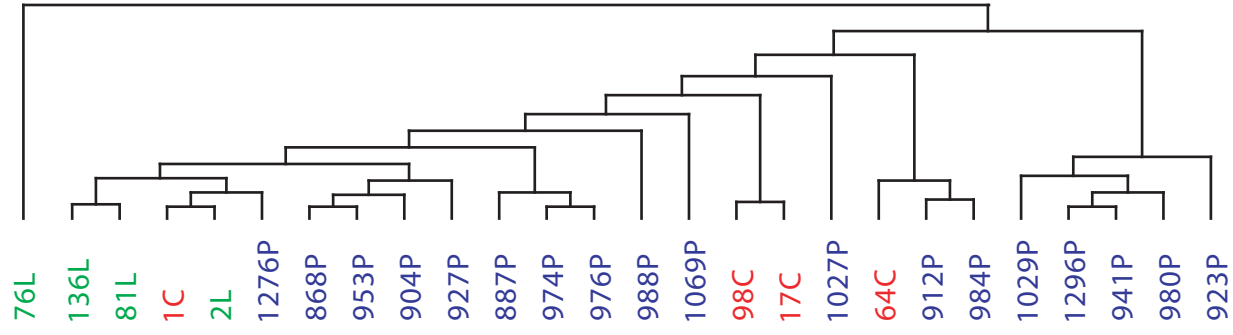

Supplement: Additional file 4 — Tumor clustering based on genes derived from cell lines modeling the metastasis process. Dendrogram from hierarchical clustering analysis of a panel of primary carcinomas (n = 18), liver metastases (n = 4), and carcinomatoses (n = 4), on genes associated with cell lines derived from tumors with different metastatic status. [file 1476-4598-6-2-S4.pdf]
